# Open Bite Classification Using Machine Learning: A Cephalometric Analysis

**DOI:** 10.3390/jcm15041494

**Published:** 2026-02-14

**Authors:** Salih Abu Shahin, Loai Abdallah, Kareem Midlej, Peter Proff, Nezar Watted, Fuad A. Iraqi

**Affiliations:** 1 Department of Clinical Microbiology and Immunology, Gray Faculty of Medicine and Health Sciences, Tel Aviv University, Tel Aviv 6997801, Israel; salih.shahin@gmail.com (S.A.S.); kareemmidlej@mail.tau.ac.il (K.M.); 2 Department of Information Systems, The Max Stern Yezreel Valley Academic College, D.N. Emek Yezreel 1930600, Israel; loaia@yvc.ac.il; 3 Department of Orthodontics, University Hospital of Regensburg, University of Regensburg, 93047 Regensburg, Germany; peter.proff@ukr.de; 4 Center for Dentistry Research and Aesthetics, Jatt 4491800, Israel; nezar.watted@gmx.net; 5 Department of Orthodontics, Faculty of Dentistry, Arab American University, Jenin 240, Palestine

**Keywords:** anterior open bite, malocclusion, skeletal patterns, vertical craniofacial, cephalometric parameters, machine learning, decision tree classifier

## Abstract

**Background**: Anterior open bite (AOB) is a complex malocclusion characterized by different vertical craniofacial growth and heterogeneous skeletal patterns, making objective diagnosis challenging using conventional cephalometric assessment alone. Recent advances in machine learning offer new opportunities to improve phenotypic characterization and diagnostic accuracy in orthodontics. **Methods**: This retrospective study analyzed lateral cephalometric records from 1056 orthodontic patients, comprising 621 patients with an anterior open bite and 435 healthy controls, all of whom were from the Arab population in Israel. Five clinically relevant cephalometric parameters related to vertical skeletal relationships were evaluated: the mandibular plane angle (ML-NSL), palatal plane angle (NL-NSL), posterior to anterior facial height ratio (PFH/AFH), gonial angle, and the facial axis. Statistical comparisons were made between the open bite and healthy subgroups, and these analyses were conducted in an exploratory framework to support hypothesis generation. A decision tree classifier was developed to distinguish AOB from healthy subjects using these features, and model performance was evaluated on a hold-out test set. Additionally, agglomerative hierarchical clustering was applied to explore latent craniofacial phenotypes. **Results**: Significant differences in vertical skeletal parameters were observed between open-bite and healthy subjects across various subgroups. The decision tree classifier achieved a test accuracy of 96.2%, with a precision, recall, and F1-score of approximately 0.97. ML-NSL emerged as the most influential feature, followed by facial axis and PFH/AFH. Unsupervised clustering identified ten distinct craniofacial clusters, including pure open bite and pure healthy phenotypes, as well as mixed clusters representing borderline or intermediate skeletal patterns. Clusters dominated by open bite cases exhibited steep mandibular planes, reduced PFH/AFH ratios, increased gonial angles, and decreased facial axis values, consistent with known vertical dysplasia patterns. **Conclusions**: Machine learning applied to cephalometric data enables accurate classification and meaningful phenotypic stratification of anterior open bite malocclusion. Beyond binary diagnosis, clustering analysis reveals clinically relevant subgroups that reflect varying degrees and types of vertical skeletal imbalance. These findings support the potential role of interpretable machine learning models as decision-support tools in orthodontic diagnosis and personalized treatment planning.

## 1. Introduction

Malocclusion, a common dental condition affecting a large portion of the population, refers to misalignment of the teeth and jaws. It can adversely impact oral functions such as chewing and speaking, complicate oral hygiene, and also influence facial aesthetics, often diminishing an individual’s self-confidence and self-esteem [1,2,3]. Among various malocclusions, an anterior open bite (AOB) is characterized by a lack of vertical overlap or contact between the upper and lower incisors when the jaws are in full closure. In practical terms, a normal overbite is about 2–3 mm, whereas an open bite presents as a negative overbite (≤0 mm) [3]. Epidemiological studies indicate that anterior open bite affects approximately 3.5% to 16.5% of the population, with prevalence varying by age and ethnicity [4,5,6]. Notably, anterior open bite can occur across virtually all traditional Angle classifications (Class I, II, III), making it a broadly relevant problem in orthodontics [7].

The etiology of anterior open bites is complex and often multifactorial. AOB has been associated with an interplay of skeletal and genetic factors, as well as environmental influences and habitual behaviors such as thumb sucking, tongue thrusting, and prolonged pacifier use, which can contribute to anterior open bite by altering the normal equilibrium of pressures on the dentoalveolar structures [3]. Skeletal hyper divergence (excessive vertical jaw growth), airway obstruction leading to mouth breathing, neuromuscular conditions, and even traumatic injuries to the jaws or temporomandibular joint have all been implicated in AOB development [3]. The condition is widely regarded as multifactorial, involving genetic regulation of craniofacial growth, skeletal development, and environmental influences, and a substantial genetic contribution to craniofacial morphology [8,9], underscoring the need for comprehensive diagnostic approaches.

Traditional approaches like Kim’s Overbite Depth Indicator (ODI) incorporate some of these measurements into a single index to assess anterior vertical dysplasia by summing the A-B plane to mandibular plane angle and the palatal plane to FH angle to quantify the degree of overbite [10]. Skeletal hyper-divergence is sometimes colloquially referred to as long face syndrome, a condition characterized by excessive vertical development of the lower third of the face, typically manifesting as a steep mandibular plane and increased anterior facial height.

Clinically, vertical skeletal parameters such as the mandibular plane angle (ML—NSL), facial height ratios (PFH/AFH), and indices like the Overbite Depth Indicator (ODI) are established diagnostic markers. This study moves beyond these standard binary indicators by utilizing machine learning to uncover latent craniofacial phenotypes and provide automated diagnostic classification.

Given the clear cephalometric distinctions of AOB, there is growing interest in applying machine learning (ML) to assist in its classification and diagnosis. Machine learning models can potentially learn complex patterns from cephalometric data that distinguish open bite cases, offering a complementary tool to orthodontic clinicians. Recent work has already integrated ML in orthodontics, for instance, to predict treatment decisions for AOB patients [7] or to improve the diagnosis of skeletal discrepancies [11]. Beyond single-condition models, the broader role of AI in orthodontics has been summarized in recent evidence syntheses. A systematic review reported expanding clinical applications of AI across orthodontic diagnosis, treatment planning, and treatment monitoring, emphasizing that these systems are intended to support, rather than replace, clinical decision-making [12]. Similarly, a scoping review highlighted that ML/DL methods are increasingly used for image-based tasks relevant to orthodontics, including automated detection of anatomical reference points and other diagnostic workflows [13]. In addition, AI tools have been explored for face-driven treatment planning, for example, AI-powered face enhancement technologies were investigated as a potential guide for improving facial harmony in orthodontic planning, albeit in a simulated/AI-generated setting [14]. However, there remains a need for research focused specifically on using ML to classify anterior open bite malocclusion. In this study, we leverage a large cephalometric dataset of patients from an Arab orthodontic population to develop an ML-based classification model for open bite and to perform an exploratory clustering of craniofacial patterns. Our aims were to (1) identify which cephalometric features most strongly differentiate AOB from normal vertical overlap, and (2) uncover distinct subgroups (phenotypes) of open bite and healthy patients via unsupervised learning. The goal is to assess the utility of machine learning in enhancing orthodontic diagnosis of open bite, and to provide insights that could inform personalized treatment planning. Recent years have seen a rapid expansion of artificial intelligence applications in orthodontics, including automated diagnosis, treatment planning, and outcome prediction using both traditional machine learning and deep learning approaches. While many of these studies focus on high-performance predictive models, concerns remain regarding interpretability and clinical transparency. The present work contributes to this growing literature by emphasizing interpretable, morphology-driven machine learning models that align closely with established orthodontic diagnostic reasoning.

## 2. Methods

This retrospective study analyzed lateral cephalometric records of 1056 orthodontic patients, 409 males and 647 females, drawn from a clinical archive of Israeli Arab patients with various malocclusions. Patients spanned the three malocclusion classes (Class I, II, III), with a wide range of ages, from children to adults. For the purposes of this study, each patient was assigned a binary label by expert orthodontists, indicating either “Open Bite” or “Healthy” (normal overbite) status. The “Open Bite” group consisted of patients with an anterior open bite (621 patients), while the “Healthy” subjects had a normal overbite and served as controls (435 patients).

### 2.1. Cephalometric Measurements

**Figure 1:**
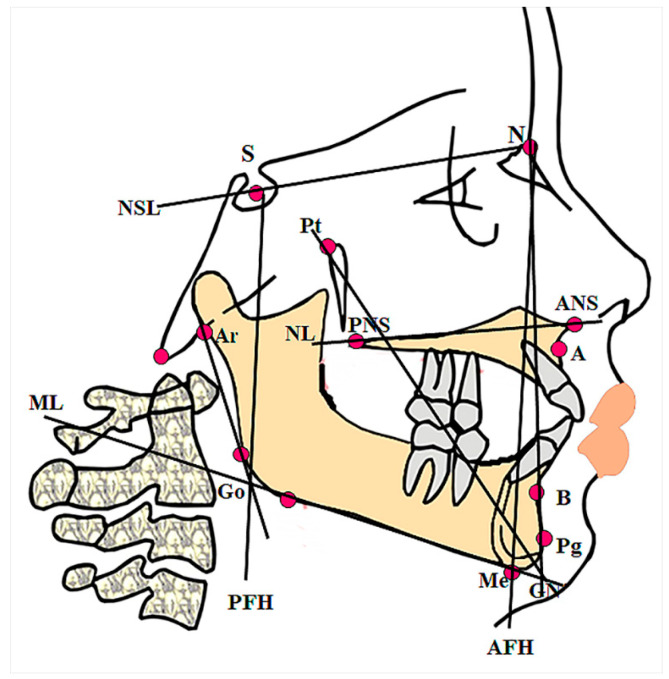
Lateral cephalometric diagram illustrating reference lines and angles. The read dots in the figure are reference points in the cephalometric image, and the lines between them provide the basis for estimating the angle between them. The figure depicts the cranial base (NSL), palatal plane (NL), and mandibular plane (ML) used to derive ML-NSL and NL-NSL. It further highlights the facial axis (from the foramen rotundum to gnathion), the gonial angle (at the junction of the ramus and mandibular body), and the linear dimensions used for the PFH/AFH ratio calculation.

A set of six key cephalometric features was extracted from each patient’s lateral cephalogram, including ML-NSL, NL-NSL, PFH/AFH, Gonial Angle, and Facial Axis, as shown in Figure 1.

The NL-ML angle was explicitly excluded only from the machine learning feature set to assess the diagnostic robustness of the remaining vertical parameters independent of this known high-performing predictor, but the NL-ML feature was retained for descriptive and inferential statistical comparisons between groups. No additional variables were explored to maintain model parsimony and focus specifically on features with established clinical relevance to vertical jaw relationships. In total, five major skeletal measurements related to vertical jaw relationships were chosen based on their established diagnostic to vertical craniofacial growth and anterior open bite [15,16]. Beyond the primary angles, other vertical diagnostics such as the facial axis and gonial angle provide insights into growth direction and mandibular rotation pattern, as described in classical craniofacial growth theory [17]. To assist in clinical interpretation, the parameters used in this study and their typical normative ranges are summarized in Table 1.

- Mandibular Plane Angle (ML-NSL): The angle between the mandibular plane (ML) and the cranial base (NSL, Nasion–Sella line). This is equivalent to the SN-GoMe angle in many analyses. Higher values indicate a steeper mandibular plane (vertical growth pattern) [15].
- Palatal Plane Angle (NL-NSL): The angle of the maxillary plane to the cranial base. This angle was also considered, as some open bite patients exhibit tipping of the palatal plane.
- Posterior Facial Height/Anterior Facial Height (PFH/AFH) Ratio: A proportional measure of vertical development. Lower ratios indicate a relatively short posterior facial height and a long anterior facial height, a hallmark of skeletal open bite.
- Gonial Angle: The angle at the junction of the ramus and mandibular body (gonion). Open bite patients often have an increased gonial angle, which is associated with a longer, narrower face.
- Facial Axis: The angle or inclination of the facial axis. A smaller facial axis angle indicates a more vertical growth direction, often seen in open bites.

These features were computed using standard cephalometric analysis software or digital tracing methods. Additionally, basic demographic data (age and sex) and malocclusion classification for each patient were recorded. Age was stratified into categories (children < 13, adolescents 14–20, adults ≥ 21) for subgroup analysis. Sex and malocclusion classes were used to examine any systematic differences in measurements.

### 2.2. Preprocessing and Statistical Analysis

Before modeling, the data was cleaned and normalized. Continuous cephalometric measures were checked for outlier samples. Outlier detection was performed using DBSCAN [18], a density-based, unsupervised algorithm designed to identify sparse observations that are weakly connected to the main data distribution. DBSCAN was selected because it does not require predefining the number of clusters and is inherently robust to moderate parameter variation, as it separates dense regions from isolated noise points. Exactly 100 cases (about 9.5% of the initial dataset) were identified as outliers, representing extreme morphological configurations relative to the dominant cephalometric patterns, and were excluded before clustering to limit the influence of noise in this exploratory analysis. Multiple independent Student’s *t*-tests, a statistical test used to test whether the difference between the responses of two groups is statistically significant or not, were performed across several predefined subgroups (e.g., age, sex, and malocclusion class) to assess differences in cephalometric measurements between open-bite and healthy subjects. Given the exploratory and hypothesis-generating nature of these subgroup analyses, no formal correction for multiple comparisons (e.g., Bonferroni or false discovery rate adjustment) was applied. Accordingly, *p*-values should be interpreted with caution and primarily as indicators of potential trends rather than confirmatory statistical evidence. Detailed preprocessing and outlier detection procedures are provided in Supplementary Methods S2.

### 2.3. Machine Learning Classification

A CART (Classification and Regression Tree) of the machine learning approach was selected for this study [19]. This algorithm was prioritized over complex black-box models like neural networks because it produces interpretable, rule-based logic (e.g., feature thresholds) that aligns with clinical diagnostic reasoning. The model utilized an entropy-based information gain criterion to determine optimal feature splits. To ensure robust evaluation, the data were partitioned into a 70% training set for model development and hyperparameter tuning, and a 30% hold-out testing set to assess generalizability on unseen data. We trained models on all possible subsets of the five features, ranging from univariate to all five, and recorded their performance, thereby identifying which combination yielded the best classification of AOB. Evaluation metrics: Model performance was evaluated on the test set using accuracy, precision, recall, and F1-score. The confusion matrix was examined to see the trade-off between false positives and false negatives.

### 2.4. Unsupervised Clustering Analysis

In addition to binary classification, we applied clustering to the cephalometric data to explore natural groupings of patients. We hypothesized that unsupervised learning might reveal clusters corresponding to degrees or subtypes of open bite vs. normal vertical patterns.

Agglomerative hierarchical clustering was applied to the five selected cephalometric variables to identify ten distinct clusters, enabling visualization of relationships between clusters via a dendrogram. For each cluster, the mean and standard deviation of each cephalometric variable were calculated to characterize cluster-specific patterns. Detailed algorithmic configurations, including distance metrics and procedures for cluster characterization, are provided in Supplementary Methods S1.

## 3. Results

### 3.1. Cephalometric Differences in Open Bite vs. Healthy

A total of 956 patients were analyzed. As expected, the open bite group showed distinct cephalometric deviations in vertical dimensions compared to healthy controls.

Although NL-ML was not included in the machine learning models, it is reported here as a conventional cephalometric reference for group comparisons and phenotype description. Between malocclusion classes, the meaning of the feature NL-ML was tested, and no significant differences were found at a 5% significance level, as shown in Table 2 and Table 3.

For all malocclusion classes, we used independent *t*-tests to compare the mean NL–ML angle between the open bite group and the healthy control group. These comparisons were performed within various subgroups defined by age category, gender, and malocclusion class. Additional *t*-test analyses were conducted on combined subgroups to ensure that any observed differences in NL–ML means were consistent across all malocclusion classes. In a class III malocclusion, a significant difference was found between males and females. In the female subgroup, a significant difference was found between malocclusion class III and class II, as well as between class III and class I, and is presented in Table 4 and Table 5.

Overall, these statistical findings establish a clear skeletal profile for AOB in our sample. These serve as a foundation for applying machine learning classification, as described next.

### 3.2. Machine Learning Classification Performance

Using the five selected cephalometric features, we trained a decision tree classifier to distinguish anterior open bite patients from healthy controls. We run several decision trees, each time choosing a subset combination from the five features. This enables us to examine the subset features that yield the best results and gain insight into the importance of each feature. The analysis of 31 feature subsets revealed a wide range of model performance. The best-performing model (utilizing NL/NSL and ML-NSL) achieved an accuracy of 96.5%, while the median accuracy across all tested combinations was 77.0% (utilizing PFH/AFH, Gonial Angle, and Facial Axis). The lowest-performing model was the univariate analysis of NL/NSL alone, which yielded an accuracy of 49.5%. Complete classification performance results across all evaluated feature subset combinations are presented in Supplementary Table S1.

We emphasize that these performance metrics are specific to our dataset; however, they suggest that an ML model can reliably classify open bite using a limited set of cephalometric measurements.

### 3.3. Clustering Analysis: Uncovering Craniofacial Pattern Subgroups

The hierarchical clustering of patients based on their cephalometric features yielded ten distinct clusters labeled 0 through 9; each cluster contains a mix of Healthy and Open Bite cases.

Table 6 summarizes the cluster profiles and the proportion of open bite vs. healthy cases in each cluster. In addition, we calculate the purity of each cluster as the maximum number of healthy and open bite samples in the cluster, divided by the total number of samples in the cluster, to describe the purity of our cluster.

By examining the class counts and purity of each cluster, we can determine which phenotype each cluster predominantly represents:

- Clusters predominantly Open Bite: Clusters 1 and 3 are 100% Open Bite (all samples are open bite, purity = 1.0). Clusters 5 (~91% open bite), 2 (~84%), 0 (~62%), 6 (~64%), and 9 (~57% open bite) also have the most Open Bite cases. These clusters are largely characterized by features associated with open bite phenotypes.
- Clusters predominantly Healthy: Cluster 4 is 100% Healthy (all samples healthy, purity = 1.0). Clusters 7 (~98% healthy) and 8 (~93% healthy) are also overwhelmingly healthy. These clusters represent normal occlusion (healthy) phenotypes.

Notably, some clusters (e.g., clusters 0, 6, 9) are mixed, with a significant proportion of both classes (purity ranging from 0.56 to 0.64). This indicates overlap in the measured features between mild open bite cases and normal cases, where individuals with similar feature values cluster together, despite differing clinical labels. Cluster 9, for example, has 38 open bites vs. 29 healthy cases (purity ~0.57), suggesting it captures individuals whose measurements are intermediate and less distinguishable by phenotype.

### 3.4. Feature Distribution Differences by Phenotype

The clustering analysis successfully stratified patients into groups that reproduce established vertical skeletal phenotypes. Clusters dominated by open bite cases (e.g., Clusters 1, 3, 5) consistently displayed hyperdivergent patterns, characterized by increased mandibular plane angles and reduced posterior to anterior facial height ratios, whereas healthy clusters showed balanced or hypodivergent growth patterns. These clusters serve as descriptive summaries of craniofacial variation and are not currently validated against clinical treatment outcomes. Detailed cluster-wise descriptive statistics for all cephalometric variables are provided in Supplementary Table S2. Subgroups and Phenotypic Variations Identified

Importantly, the unsupervised clustering not only distinguished healthy vs. open bite individuals, but also revealed subgroups that likely correspond to different severities or morphological subtypes within those categories:

- Among the Open Bite clusters, we see multiple clusters that all comprise open bite cases but differ in their feature averages. For example, Cluster 1 vs. Cluster 3. Both are pure open bite clusters; yet cluster 1 has more extreme vertical features, with the highest NL/NSL and ML-NSL, and a very low PFH/AFH, compared to cluster 3, which, while still vertical, is slightly less extreme. This suggests cluster 1 represents a “severe vertical growers” open bite subgroup, whereas cluster 3 might represent a moderate open bite pattern. Likewise, Cluster 5 (90% open bite) shares the high angles, NL/NSL ≈10°, ML-NSL ≈43°, and low PFH/AFH (~60%), typical of vertical growers, but its gonial angle (~130.5°) is noticeably lower than clusters 1 and 3, which are ~138–141°. This could indicate cluster 5 represents an open bite subtype with a slightly different jaw morphology (e.g., open bite with a less obtuse gonial angle). Meanwhile, clusters like 2, 6, 9, which have a mix of open bite and some healthy cases, correspond to milder open bite cases or borderline phenotypes that overlap with normal craniofacial measurements. For instance, cluster 9’s open bite individuals have nearly normal NL/NSL and facial axis values, suggesting that these may be anterior open bites of dental origin or minimal skeletal divergence, clustering together with healthy individuals who share similar skeletal measurements.
- Among the Healthy clusters, we also observe variations. Cluster 4 stands out as a cluster of entirely healthy individuals with very low vertical proportions, the highest PFH/AFH ratio (~73%), and the lowest ML-NSL angle (~26°) of all clusters. These could be “low-angle” normal cases, short-face, possibly even deep bite tendency. In contrast, Cluster 7 (98% healthy) has an unusually high mean NL/NSL (~10°) combined with average ML-NSL (~32°) and PFH/AFH (~68%). This might represent healthy individuals with a slightly steeper palatal plane or mild hyperdivergent tendencies who nonetheless do not exhibit an open bite. Cluster 8 (93% healthy) has a high gonial angle (~133°), comparable to open bite levels but also the highest facial axis (~95.7°) and a moderate ML-NSL (~30.6°). This combination suggests cluster 8 could correspond to healthy subjects with larger jaw angles but otherwise horizontally oriented growth, perhaps compensation that prevented an open bite. In summary, the healthy phenotype is not homogeneous; the clustering hints at different normal occlusion morphologies (e.g., low-angle vs. high-angle normals), all of which maintain an overall balance that avoids the open bite condition.

### 3.5. Conclusion and Implications

In this study, we have demonstrated that clustering analysis effectively separates the dataset into meaningful groups that correspond to the two clinical phenotypes: open bite versus normal, as evidenced by the high-purity clusters for each class and the clear differences in feature averages. Clusters 1, 3, 5, and others represent the open bite phenotype; these clusters align with open bite patients. In contrast, clusters 4, 7, and 8 represent the healthy phenotype, characterized by a normal occlusion. The cluster labels and their class compositions make it straightforward to identify which clusters are associated with open bite versus healthy individuals.

Moreover, the clustering results are consistent with known cephalometric characteristics of these phenotypes. Open bite clusters show the classic vertical growth pattern, whereas healthy clusters exhibit more favorable proportions. This agreement with orthodontic research lends credibility to clustering; it has essentially rediscovered the key skeletal differences between open-bite and normal-bite groups without supervision.

Finally, the clustering has also identified subgroups within the open bite and healthy categories, suggesting that there are nuanced differences in severity or morphology that the model has picked up. For example, the open bite cases naturally fell into distinct clusters corresponding to the severity or which skeletal dimensions were most pronounced in their malocclusion, and the healthy cases also showed variability that was captured in separate clusters. These insights could be valuable for further analysis, for example, tailoring treatment approaches to specific open bite subtypes or understanding normal variation. The presence of mixed clusters, such as cluster 9 and 0, highlights an overlap zone where some open bite individuals are skeletal borderline, hence their measurements approach normal ranges. Indicating those cases might require different diagnostic consideration.

In conclusion, cluster analysis has proven to be a useful tool in differentiating Open Bite vs. Healthy phenotypes. We identified the clusters that correspond to each phenotype and confirmed that the clustering outcome reflects real anatomical differences, as supported by the literature. The clusters representing open bite versus healthy were clearly identified, and the feature profiles of these clusters validate the known characteristics of each phenotype [20]. The results not only stratify the two groups but also provide a deeper understanding of intra-group variability, different “types” of open bite, and normal cases, which can be further explored for clinical implications. Overall cluster purity values and the descriptive statistics suggest that the clustering is largely successful in capturing the underlying phenotype distinctions in the data. This unsupervised approach complements binary classification by revealing underlying subgroups that a simple ‘healthy/open bite’ label might obscure.

## 4. Discussion

In this study, we applied machine learning techniques to classify and characterize anterior open bite malocclusion using lateral cephalometric measurements. The results demonstrated that even a relatively simple ML model, a decision tree, can achieve very high accuracy in identifying AOB patients based on key skeletal features. The findings also highlighted distinctive subgroups of vertical craniofacial morphology through clustering. Here, we discuss the implications of these results in the context of orthodontic diagnosis, compare them with existing literature, and outline potential clinical and research significance.

### 4.1. Skeletal Diagnostics of AOB

Our analysis reaffirmed that certain cephalometric indicators carry strong diagnostic weight for anterior open bite. Chief among these is the mandibular plane angle (ML-NSL). Practitioners have long recognized that AOB correlates with a steep mandibular plane and a “hyperdivergent” facial pattern [20,21], sometimes colloquially referred to as a long-face syndrome. Additionally, we observed significantly lower posterior-to-anterior facial height ratios in open bite cases, which is consistent with the Jarabak analysis of vertical dysplasia, Jarabak’s ratio decreases as the bite opens [21]. This aligns with Kim’s classic Overbite Depth Indicator concept, where a combination of steep mandibular plane and increased lower anterior face height flags an open bite tendency [10]. Our decision tree’s primary split on ML-NSL, at ~35°, effectively operationalized this concept. Interestingly, our data suggests a threshold in the mid-30s degrees for ML-NSL, above which patients are very likely to have an open bite.

The gonial angle emerged as another important feature. Patients with open bites had gonial angles often above 130°, reflecting a backwards-rotated mandible. This finding is supported by previous studies describing open bite craniofacial patterns. Previous studies have noted that dolichofacial, long-face individuals tend to exhibit larger gonial angles due to the downward rotation of the mandibular ramus [22]. Our cluster analysis even identified a subset (cluster 5) where gonial angle was disproportionately high. This pattern corresponds to one of the skeletal open bite subtypes previously described using individualized cephalometric classification [21,23]. They delineated skeletal open bite with a reduced ML-NSL angle as a distinct category, meaning that some open bite patients do not have an extreme mandibular plane angle but do exhibit other skeletal deviations, such as a large gonial angle or upper/lower jaw inclination issues. Cluster 5 in our results fits this description well; those patients likely represent the less common subtype where the open bite etiology might be more related to jaw shape or dentoalveolar factors than a truly high-angle mandible. The existence of such subtypes is clinically important, as presented in Table 7. It suggests that not all anterior open bites are due to the same skeletal pattern. Some patients might appear relatively average in mandibular plane inclination yet still present an open bite. This finding may be explained by either bone adaptation around the teeth (alveolar compensation) or the tongue being habitually positioned (tongue posture), and it might be overlooked if one relies solely on a single measurement.

### 4.2. Role of Machine Learning in Diagnosis

The high performance of the decision tree (≈96.5% accuracy) indicates that ML can reliably capture the relationships between these cephalometric measurements and the presence of open bite. This level of accuracy is comparable to or even superior to previous machine learning efforts in orthodontic classification tasks. For instance, [24] developed a diagnostic model for skeletal open bite and reported about 93% accuracy using a combination of seven cephalometric variables. Our model achieved better accuracy with a smaller number of variables, reinforcing that a well-chosen set of measurements contains rich diagnostic information. One advantage of our approach is interpretability, the resulting decision rules (e.g., “if ML-NSL > X and PFH/AFH < Y, then Open Bite”) can be directly interpreted by clinicians and even used as a simple checklist in practice. Interpretability is especially important in clinical settings, survey work highlights the need for transparent, explainable machine learning to enhance clinical trust and usability [25]. This emphasis aligns with contemporary orthodontic AI reviews, which describe rapid growth in AI applications while reinforcing the continued need for clinically interpretable and responsibly validated decision-support tools [12,13]. Such transparency is often limited in more complex black-box models, including many neural network-based approaches. Similar interpretable machine learning frameworks have been applied to analyze craniofacial anatomical relationships in an interpretable way [11].

It is worth noting that in [7], a recent study applying ML to AOB cases, more complex algorithms such as CNNs and Random Forests reached ~83% accuracy in predicting treatment decisions for open bite [7]. While that study had a different goal, it underscores the growing application of ML in orthodontics and the capability of these models to achieve high accuracy. Our results focused on diagnosis and classification rather than treatment, and the fact that even an easily implementable decision tree outperformed some advanced models is encouraging. It suggests that for certain well-defined problems, such as distinguishing between open bite and normal, simpler ML models suffice and can even be preferable due to their clarity. In a clinical setting, an orthodontist could input a patient’s cephalometric measurements into a decision-tree-based calculator and get an immediate, explainable risk assessment for skeletal open bite. This model may serve as an interpretability-focused decision-support tool for clinicians assessing borderline skeletal discrepancies. However, the current results are subject to potential overfitting to the regional dataset, and future studies must provide external validation on diverse populations before clinical implementation.

### 4.3. Insights from Clustering

The unsupervised clustering of cephalometric data yielded ten distinct patient subgroups (Clusters 0 through 9), each corresponding to a particular vertical growth pattern or anterior open bite severity. Notably, these data-driven clusters align well with known clinical phenotypes, ranging from severe skeletal open bites to healthy cases. For clarity, the clusters can be broadly grouped as follows: severe skeletal open bite clusters (Clusters 1 and 3), moderate open bite clusters (Clusters 2 and 5), healthy clusters (Clusters 4, 7, and 8), and mixed or borderline phenotype clusters (Clusters 0, 6, and 9). Each subgroup exhibits characteristic cephalometric features with important implications for diagnosis and treatment planning, as detailed below.

### 4.4. Severe Skeletal Open Bite Subgroups (Clusters 1 and 3)

Clusters 1 and 3 consist exclusively of anterior open bite patients (0 healthy cases in each cluster, purity 100%), representing the most severe skeletal open bite phenotypes in our sample. Cephalometrically, these clusters are characterized by extreme vertical dysplasia: patients in Clusters 1 and 3 exhibited the lowest PFH/AFH ratios among all clusters, indicating a markedly reduced posterior facial height relative to anterior facial height. Such low ratios (on the order of ~0.55, i.e., ~55%) fall well below the diagnostic threshold [20]. In addition, these severe open bite clusters showed steep mandibular plane angles, high ML-NSL, and obtuse gonial angles, reflecting a pronounced clockwise rotation of the mandible. The facial axis in these patients was correspondingly reduced, consistent with a vertical growth direction. These cephalometric findings, decreased PFH/AFH and increased vertical angles, are hallmark features of skeletal open bite patterns [20]. Clinically, patients in Clusters 1 and 3 represent prototypical high-angle, long-face cases with severe anterior open bites. Such individuals often require more aggressive treatment modalities focused on vertical control. For growing patients, this might involve high-pull headgear or vertical-pull chin cups to restrain vertical growth, and for adults, treatment often necessitates orthognathic surgery (e.g., superior repositioning of the maxilla or mandibular clockwise rotation) or skeletal anchorage to intrude molars. The clear delineation of these severe open bite cases by the clustering algorithm highlights its utility. It automatically identifies patients with the most extreme vertical dysplasia, who may benefit from early intervention and careful monitoring of vertical growth to prevent bite opening.

### 4.5. Moderate Open Bite Subgroups (Clusters 2 and 5)

Clusters 2 and 5 also predominantly comprise open bite cases (approximately 84–91% of cases in these clusters were open bite), but unlike Clusters 1 and 3, they include a small minority of healthy cases. These clusters appear to represent moderate skeletal open bite phenotypes, characterized by patients with an intermediate severity of anterior open bite malocclusion. The cephalometric features of Clusters 2 and 5 are correspondingly less extreme than those of Clusters 1 and 3. For instance, the PFH/AFH ratios were moderately reduced in these groups, reflecting a mild to moderate imbalance in vertical facial proportions. The mandibular plane (ML-NSL) angles and gonial angles were elevated but not as pronounced as in the severe open bite clusters. In other words, patients in Clusters 2 and 5 still show a hyperdivergent growth pattern, but to a lesser degree than those in Clusters 1 and 3. Clinically, this suggests that these moderate open bite cases may be manageable with conventional orthodontic mechanics supplemented by skeletal anchorage, without always resorting to orthognathic surgery. For example, vertical control can be achieved via posterior tooth intrusion or anterior extrusion, combined with habit control or myofunctional therapy if etiologic factors are contributing. The identification of moderate open bite subgroups is valuable for tailoring treatment planning, it indicates that not all open bites are identical, and those in Clusters 2 and 5 might be amenable to less invasive treatment approaches compared to the more extreme cases in Clusters 1 and 3. By stratifying open bite patients into severe and moderate categories, the clustering provides a nuanced diagnostic aid. It helps clinicians predict which cases might respond to orthodontic camouflage alone versus which will likely require combined surgical-orthodontic intervention.

### 4.6. Healthy Subgroups (Clusters 4, 7, 8)

Three clusters (Clusters 4, 7, and 8) were composed almost entirely of healthy individuals, corresponding to normal or deep-bite vertical phenotypes. Clusters 7 and 8 contained 98% and 93% healthy cases, respectively, while Cluster 4 was 100% healthy. The existence of multiple healthy clusters suggests that the algorithm detected subtle heterogeneity within the control group, likely separating individuals with normal vertical dimensions from those with a tendency toward deep bite and hypodivergent growth. For example, Cluster 4, a smaller cluster of 51 healthy subjects and zero open bites, may represent an extremely hypodivergent subgroup. Indeed, patients in Cluster 4 exhibited cephalometric traits opposite those in the open bite groups, characterized by high PFH/AFH ratios [20], as well as low mandibular plane angles and acute gonial angles. Such a combination is indicative of a short lower face height and a tendency towards a deep bite. Clinically, individuals in this cluster may present with characteristics such as a deep overbite, a low mandibular plane, and strong jaw musculature, and they may be prone to developing deep bite malocclusions or temporomandibular joint stress due to their jaw geometry. In contrast, Clusters 7 and 8 are likely to correspond to more average norms within healthy variation; their cephalometric means would be closer to standard values. These two clusters may differentiate between slight variations, such as one exhibiting a “neutral-normal” vertical pattern and another a mildly hypodivergent pattern, although both fall within acceptable ranges. The separation of healthy subjects into distinct clusters highlights that even normal occlusions encompass a spectrum of vertical craniofacial patterns, ranging from average to mildly deep-bite configurations. From a clinical standpoint, recognizing these subgroups among ostensibly normal patients can be useful. It alerts the clinician to patients who, despite having no open bite, may have a propensity for a deep bite or reduced lower face height, which could influence treatment decisions. For instance, an orthodontist might choose mechanics that avoid excessive bite deepening in a hypodivergent patient (Cluster 4) or be mindful of retaining the vertical dimension during retention, thus preventing relapses toward deep bite. In summary, Clusters 4, 7, and 8 demonstrate that the clustering approach can differentiate normal versus short-face phenotypes, providing a more granular classification of healthy individuals than a one-size-fits-all normal category.

### 4.7. Mixed/Borderline Phenotype Subgroups (Clusters 0, 6, 9)

Clusters 0, 6, and 9 have a mixed composition of healthy and open bite cases, with neither phenotype overwhelmingly dominant; open bite cases comprise roughly 57–63% of each cluster. These clusters seem to represent borderline or intermediate phenotypes, patients whose vertical skeletal pattern is not clearly normal or open bite, but somewhere in between. The cephalometric features of these groups tend to be intermediate as well. For instance, the average PFH/AFH ratios in Clusters 0, 6, and 9 are around the neutral range, approximately 0.60–0.62, falling between the low values of the open bite groups and the high values of the deep-bite group. Similarly, the ML-NSL angles and other vertical measurements in these clusters are mildly elevated relative to normal, but significantly lower than those observed in the frank open bite clusters. In practical terms, patients in Clusters 0, 6, and 9 might present with only a slight anterior open bite or open bite tendency, or with vertical skeletal discrepancies that are borderline (e.g., a long face tendency without an obvious anterior open bite, or an open bite that is primarily dental/alveolar in nature rather than severe skeletal). Cluster 9, for example, contained 38 open bites vs. 29 healthy cases, nearly a fifty-fifty split, suggesting it may encapsulate cases at the threshold of an open bite diagnosis (e.g., minimal incisor separation or open bite that becomes apparent only under certain conditions). From a clinical perspective, these borderline clusters are particularly important because they highlight the continuum of vertical malocclusion. They reinforce that an anterior open bite is not a binary condition but exists on a spectrum. Patients in these intermediate clusters might easily be overlooked in a binary classification. However, the cluster analysis flags them as a distinct group, which could benefit from early intervention or careful observation. For instance, a child with a borderline open bite might not yet show severe symptoms, but the clustering suggests their growth pattern is trending toward open bite. This could prompt the clinician to implement habit control, if thumb sucking or tongue thrust is present, or use growth-modification appliances to prevent progression. In adults, borderline cases might be managed with minor orthodontic mechanics such as extrusion of incisors or minor posterior intrusion rather than the more aggressive approaches needed for Clusters 1 and 3. Importantly, the identification of mixed clusters (0, 6, 9) by the AI clustering model provides a nuanced diagnostic tool. It helps in recognizing patients who lie near the diagnostic cutoff between normal and open bite. This can inform more personalized treatment planning, ensuring that borderline cases receive appropriate attention, neither over-treatment of what is essentially a mild variation, nor under-treatment that allows a developing open bite to worsen.

### 4.8. Clinical Implications of the Clustering Approach

Overall, the clustering-derived subgroups demonstrate clear clinical relevance, as they stratify patients according to the severity and nature of their vertical growth patterns. In practice, this means that an orthodontist could use such a data-driven model as a decision support tool in diagnosis for a new patient’s cephalometric data; the algorithm could assign the patient to the closest cluster, thereby revealing if they resemble a known severe open bite subgroup, a moderate subgroup, or a borderline pattern. This information supplements the traditional diagnosis by quantifying how “extreme” or “intermediate” a patient’s skeletal open bite tendency is, which is crucial for planning treatment. For example, being identified as a Cluster 1/3 patient would immediately signal a high-angle case likely needing comprehensive vertical control and possibly surgery, whereas a Cluster 5 assignment might indicate that conventional intrusion mechanics could suffice. Similarly, classification into a healthy cluster, such as 7 or 8, would alert the clinician to monitor for excessive overbite during treatment. The use of AI clustering in this way could enhance personalized treatment planning. By recognizing the specific phenotype subgroup of the patient, clinicians can tailor mechanics, predict challenges, and counsel patients on the expected treatment complexity and stability. In summary, the unsupervised clustering approach not only corroborates known cephalometric distinctions (open bite vs. healthy skeletal patterns) but also provides a more refined, continuum-based classification. Embracing such clustering in orthodontic practice or future AI-driven cephalometric analysis tools could enhance diagnostic accuracy and aid in formulating targeted treatment plans, ultimately contributing to improved management of vertical malocclusions.

Limitations: Despite the strengths of our large sample and integrated approach, several limitations are worth acknowledging. Our study focused on a specific population, Arab patients, and the cephalometric norms and prevalence of AOB can vary across ethnic groups. The model and thresholds we derived might need recalibration for other populations with different craniofacial morphologies (e.g., European vs. Asian cephalometric norms differ slightly in mandibular plane angles). External validation or k-fold cross-validation was not performed. However, the results remain meaningful for identifying internal morphological patterns and interpretable decision rules within the analyzed cohort. Future studies using independent datasets will further assess generalizability. Moreover, the classification was binary, open vs. not open bite, we did not distinguish between dental and skeletal open bites in labeling, although our clustering indirectly addressed this distinction. In practice, an orthodontist would want to know if an open bite is primarily dental, which has a better prognosis for simple treatment or skeletal, which is more challenging. Future work could incorporate that distinction into model training. Additionally, multiple subgroup comparisons were conducted using independent *t*-tests without formal correction for multiple testing. While this approach is acceptable in exploratory analyses aimed at pattern discovery, it increases the risk of type I error. Therefore, the statistical findings from subgroup analysis should be interpreted cautiously and warrant confirmation in future hypothesis-driven studies.

Another limitation is that we used a static dataset with cross-sectional cephalometric. Longitudinal changes (i.e., growth, treatment outcomes) were not analyzed. It would be valuable to determine if the clusters we found have different stability outcomes, for example, do patients in cluster 1 have more stable treatment results than those in cluster 2 after 5 years? Long-term follow-up data could enhance the model by predicting not just the classification but also the likely stability or relapse potential, which is a major concern in open bite treatment.

### 4.9. Future Directions

This work opens several avenues for future research and improvement: (1) Deep Learning and Imaging: While we used to measure cephalometric features, an alternative approach is to feed the cephalometric radiograph itself into a deep learning model, such as a convolutional neural network (CNN). Recent advances in computer vision may enable the automatic detection of open bites from cephalometric images without the need for landmarking. CNNs might capture subtle patterns, such as incisor spacing and jaw curvature, beyond linear measurements. Incorporating 2D radiographic images and even 3D imaging could improve diagnostic accuracy and provide a more holistic analysis. For instance, a 3D analysis might consider transverse dimensions and occlusal contacts, which are lost in 2D cephalograms. (2) Expanded Feature Set: We could incorporate dental measurements such as incisor angulations or molar heights, and patient habits into the model. An ML model that considers skeletal, dental, and behavioral data may predict the presence of an open bite or its severity on a continuous scale. This might also help differentiate skeletal AOB vs. dental AOB automatically. (3) Larger and Diverse Datasets: Validating the model on larger datasets from different regions would be important to ensure generalizability. If similar performance is seen in, say, a European cohort, it strengthens the case for universal application of such ML tools. Conversely, differences might reveal new insights, for example, in some populations, PFH/AFH may be less predictive due to differing vertical proportions, which could lead to customized models. (4) Integration into Clinical Workflow: Future studies could focus on the practical aspect, developing a user-friendly software or app for orthodontists that implements our trained model. A prospective study could then be done where orthodontists use the ML tool during diagnosis, and we measure if it improves their diagnostic agreement or confidence, especially among less experienced clinicians. (5) Predictive Modeling: Beyond classification, another direction is to use ML to predict treatment outcomes for open bite patients. For example, given a patient’s cluster/profile, can we predict whether they will be successfully treated with orthodontics alone or require surgery, and what the relapse risk is? Incorporating the clustering approach, one might find that patients in certain clusters have higher relapse rates, which can inform retention strategies.

Finally, further investigation into the etiological differences between clusters could be insightful. For instance, do cluster x patients have a higher incidence of certain habits or genetic markers compared to cluster y patients? Such questions bridge the gap between data-driven clusters and real causal factors.

## 5. Conclusions

In conclusion, this study demonstrates that open bite malocclusion can be effectively classified and analyzed using machine learning on cephalometric data. The introduction of a decision tree model achieved an accuracy of approximately 96.5% in distinguishing between patients with anterior open bite and healthy individuals, underscoring the diagnostic power of a few key skeletal measurements. We found that open bite patients consistently exhibit a steeper mandibular plane, larger gonial angle, and reduced posterior facial height ratio, features that a machine learning model can leverage to make accurate predictions. The decision tree’s explicit rules closely mirrored clinical reasoning, providing an interpretable decision support tool. Moreover, our unsupervised clustering revealed that the category of open bite is not monolithic; there are varying degrees and patterns of skeletal open bite. We identified clusters ranging from mild to severe hyperdivergent cases, as well as normal and deep-bite patterns. Recognizing these distinct subgroups has practical implications, as it allows for more personalized treatment planning and counseling. For example, a patient falling into an extreme open bite cluster might be advised about the likely need for orthognathic surgery and vigilant retention, whereas a patient with a mild cluster might be managed with orthodontic appliances and habit control alone.

## Figures and Tables

**Table 1:** Summary of Cephalometric Parameters and Normative Ranges.

Abbreviation	Definition	Normal Range/Value	Clinical Significance
ML-NSL	Mandibular Plane Angle	26–32°	High values indicate vertical growth
NL-NSL	Palatal Plane Angle	6–10°	Indicates inclination of the maxilla
PFH/AFH	Facial Height Ratio	>65%	<62% indicates hyperdivergent/long face
Gonial Angle	Angle of Mandible	120–130°	Larger angles indicate backward rotation.
Facial Axis	Growth Axis Angle	~90°	<90° indicates vertical growth tendency

**Table 2:** The mean, standard deviation (std), number of samples, and the standard error (se) of the NL-ML feature in Class I occlusion and malocclusion classes I and II.

Skeletal Class	Mean	Std	Number of Samples	Se
I	29.47	5.49	262.0	0.34
II	28.94	5.50	330.0	0.30
III	29.36	5.61	364.0	0.29

**Table 3:** The table presents the results of the statistical *t*-test comparing the differences in the NL-ML means between the malocclusion classes. The results show that there are no significant differences between the groups.

Malocclusion Compared Groups	Statistics *t*-Value	*p*-Value
(class I) vs. (class II)	t=1.17	p=0.242
(class I) vs. (class III)	t=0.239	p=0.811
(class II) vs. (class III)	t=−1.006	p=0.315

**Table 4:** Independent *t*-test analyses were performed to compare the means of the NL_ML angle between subgroups in the open bite group and the healthy control group to test any observed differences.

Compared Groups	Statistics *t*-Value	*p*-Value
(class III_Female) vs. (class III_Male)	t=1.701	p=0.09
(class III_Female) vs. (class II_Female)	t=1.848	p=0.065
(class III_Female) vs. (class II_Male)	t=2.018	p=0.045
(class III_Female) vs. (class I_Female)	t=2.221	p=0.027
(class III_Female) vs. (class I_Male)	t=2.728	p=0.007
(Female_0 < Age < 13) vs. (Female_Age > 21)	t=−2.061	p=0.04
(Female_14 < Age < 20) vs. (Female_Age > 21)	t=−2.877	p=0.004
(Female_Age > 21) vs. (Male_0 < Age < 13)	t=2.501	p=0.013
(Female_Age > 21) vs. (Male_14 < Age < 20)	t=1.995	p=0.047
(Female_Age > 21) vs. (Male_Age > 21)	t=1.95	p=0.053
(ClassIII_14 < Age < 20) vs. (ClassII_14 < Age < 20)	t=3.028	p=0.003
(ClassIII_Age > 21) vs. (ClassII_14 < Age < 20)	t=2.786	p=0.006
(ClassII_0 < Age < 13) vs. (ClassII_14 < Age < 20)	t=2.694	p=0.008
(ClassII_14 < Age < 20) vs. (ClassII_Age > 21)	t=−3.237	p=0.001
(ClassII_14 < Age < 20) vs. (ClassI_14 < Age < 20)	t=−2.683	p=0.008
(ClassII_14 < Age < 20) vs. (ClassI_Age > 21)	t=−2.317	p=0.022
(0 < Age < 13) vs. (Age > 21)	t=−1.813	p=0.07
(14 < Age < 20) vs. (Age > 21)	t=−2.091	p=0.037
(Open Bite _Female_0 < Age < 13) vs. (Open Bite_male_0 < Age < 13)	t=2.139	p=0.040

**Table 5:** Independent *t*-test analyses were performed to compare the means of the NL_ML angle between different malocclusion classes in the open bite group and the healthy control group to test any observed differences.

Subgroup	Mean of (NL-ML)	Std (NL-ML)	*t*-Value	*p*-Value
Healthy Class I	24.2	2.7	t=−23.26	p=0.000
Open Bite Class I	33.2	3.6		
Healthy Class II	23.7	2.6	t=−26.763	p=0.000
Open Bite Class II	32.8	3.6		
Healthy Class III	23.6	2.7	t=−27.5	p=0.000
Open Bite Class III	32.9	3.7		

**Table 6:** Summary of the cluster profiles and the proportion of open bite vs. healthy cases in each cluster. In addition, we calculate the purity of each cluster as the maximum number of healthy and open bite samples in the cluster, divided by the total number of samples in the cluster, to describe the purity of our cluster.

Cluster Number	Number Healthy Samples	Number Open Bite Samples	Purity of the Cluster
0	76	125	0.62
1	0	97	1
2	15	81	0.84
3	0	68	1
4	51	0	1
5	7	70	0.91
6	48	84	0.64
7	92	2	0.98
8	68	5	0.93
9	29	38	0.57

**Table 7:** Synthesis of Skeletal Phenotypes and Clinical Interpretation.

Phenotype Category	Key Cephalometric Features	Clinical Interpretation & Treatment Insight
Severe Skeletal AOB	Extremely high ML-NSL; PFH/AFH ~55%.	High-angle, long-face, likely requires surgery or skeletal anchorage
Moderate AOB	Elevated ML-NSL, moderately reduced PFH/AFH.	Manageable with conventional mechanics or posterior intrusion
Borderline/Mixed	Intermediate angles, PFH/AFH 60–62%.	Cases at diagnostic threshold, may be dental or minimal skeletal divergence
Healthy/Hypodivergent	Low ML-NSL, high PFH/AFH (>70%).	Deep-bite tendency, strong jaw musculature, requires bite depth monitoring.

## Data Availability

The data supporting the findings of this study are not publicly available due to ethical and privacy restrictions, but may be made available from the corresponding author upon reasonable request and subject to institutional approval.

## Supplementary Materials

The following supporting information can be downloaded at: https://www.mdpi.com/article/10.3390/jcm15041494/s1, Table S1: Decision tree classification performance across all evaluated feature subset combinations, including accuracy, precision, recall, and F1-score, Table S2: Descriptive statistics (mean and standard deviation) of the five cephalometric variables (ML-NSL, NL-NSL, PFH/AFH, gonial angle, facial axis) for each of the ten clusters identified by hierarchical clustering, Methods S1: Detailed description of the unsupervised clustering configuration, including distance metrics, linkage criteria, and cluster characterization procedures; Methods S2: Data Preprocessing and Outlier Detection.
