# Supplementary material for: Open Bite Classification Using Machine Learning: A Cephalometric Analysis
Source: J Clin Med. 2026 Feb 14;15(4):1494. doi: 10.3390/jcm15041494 (PMC12942192; doi:10.3390/jcm15041494)
Supplement: Supplementary file 1 [file jcm-15-01494-s001.zip › jcm-4092299-supplementary.pdf]

Supplementary Materials:

Supplementary **Table S1:** Decision tree classification performance across all evaluated feature subset combinations, including accuracy, precision, recall, and F1-score.

Recall	Precision	Accuracy	f1_score	Num Features	Features
0.971	0.971	0.965	0.971	2	['NL/NSL', 'ML-NSL']
0.971	0.965	0.962	0.968	5	['NL/NSL', 'PFH/AFH', ' GONIAL ANGLE', 'facial Axis', 'ML-NSL']
0.965	0.971	0.962	0.968	3	['NL/NSL', 'facial Axis', 'ML-NSL']
0.959	0.970	0.958	0.965	4	['NL/NSL', 'facial Axis', ' GONIAL ANGLE', 'ML-NSL']
0.959	0.965	0.955	0.962	3	['NL/NSL', Gonial Angle, 'ML-NSL']
0.953	0.970	0.955	0.962	4	['NL/NSL', 'PFH/AFH', 'facial Axis', 'ML-NSL']
0.953	0.964	0.951	0.959	4	['NL/NSL', 'PFH/AFH', ' GONIAL ANGLE', 'ML-NSL']
0.953	0.964	0.951	0.959	3	['NL/NSL', 'PFH/AFH', 'ML-NSL']
0.871	0.876	0.850	0.874	4	['NL/NSL', 'PFH/AFH', ' GONIAL ANGLE', 'facial Axis']
0.830	0.850	0.812	0.84	3	['NL/NSL', 'PFH/AFH', 'facial Axis']
0.819	0.833	0.794	0.826	1	['ML-NSL']
0.807	0.836	0.791	0.821	2	['facial Axis', 'ML-NSL']
0.801	0.835	0.787	0.818	3	['PFH/AFH', 'facial Axis', 'ML-NSL']
0.784	0.854	0.791	0.817	3	['NL/NSL', ' GONIAL ANGLE', 'facial Axis']
0.807	0.826	0.784	0.817	3	[' GONIAL ANGLE', 'facial Axis', 'ML-NSL']
0.842	0.787	0.770	0.814	3	['PFH/AFH', 'Gonial Angle', 'facial Axis']
0.795	0.824	0.777	0.810	2	['PFH/AFH', 'ML-NSL']
0.795	0.810	0.767	0.802	4	['PFH/AFH', ' GONIAL ANGLE', 'facial Axis', 'ML-NSL']
0.795	0.805	0.763	0.800	2	[' GONIAL ANGLE', 'ML-NSL']
0.801	0.797	0.760	0.799	3	['NL/NSL', 'PFH/AFH', ' GONIAL ANGLE']
0.795	0.800	0.760	0.798	2	[' GONIAL ANGLE', 'facial Axis']
0.784	0.802	0.756	0.793	2	['PFH/AFH', 'facial Axis']
0.784	0.793	0.749	0.788	2	['NL/NSL', 'PFH/AFH']
0.778	0.778	0.735	0.778	3	['PFH/AFH', ' GONIAL ANGLE', 'ML-NSL']
0.760	0.783	0.732	0.772	2	['NL/NSL', 'facial Axis']
0.749	0.766	0.714	0.757	2	['PFH/AFH', ' GONIAL ANGLE']
0.725	0.785	0.718	0.754	1	['facial Axis']
0.737	0.737	0.686	0.737	1	['PFH/AFH']
0.632	0.711	0.627	0.669	1	[' GONIAL ANGLE']
0.614	0.700	0.613	0.654	2	['NL/NSL', ' GONIAL ANGLE']
0.532	0.583	0.495	0.557	1	['NL/NSL']

Supplementary Methods S1: Detailed description of the unsupervised clustering configuration, including distance metrics, linkage criteria, and cluster characterization procedures. Unsupervised clustering was performed using agglomerative hierarchical clustering. Prior to clustering, all cephalometric variables were standardized using z-score normalization to ensure comparable scaling across measurements. Pairwise distances between observations were computed using the Euclidean distance metric. Clusters were then formed using Ward's linkage criterion, which minimizes the total within-cluster variance at each agglomeration step and is well suited for identifying compact, spherical clusters in continuous data. The resulting

hierarchical structure was visualized using a dendrogram, and the final number of clusters was determined by selecting an appropriate cut level based on the dendrogram structure. We examined the composition of each cluster in terms of class label and the percentage of cluster members who were Open Bite versus Healthy, to assess cluster "purity" with respect to the known classification. We also noted any demographic biases in clusters (e.g., if a cluster contained predominantly young patients or a particular sex). Detailed cluster profiles were then formulated, describing the distinctive cephalometric signature of each cluster. These profiles were compared with known cephalometric patterns from literature. All statistical analyses were performed with Python using libraries such as NumPy, SciPy, and scikit-learn, and results were tabulated for reporting. This study was conducted under appropriate ethical guidelines, with patient records anonymized and only aggregate data reported.

Supplementary **Table S2:** Descriptive statistics (mean and standard deviation) of the five cephalometric variables (ML-NSL, NL-NSL, PFH/AFH, gonial angle, facial axis) for each of the ten clusters identified by hierarchical clustering.

Cluster	NL-NSL(°)	PFH/AFH(%)	Gonial Angle(°)	Facial Axis(°)	ML-NSL(°)
Cluster 0	8.8 ± 3.0	61.3 ± 2.7	133.4 ± 3.8	85.4 ± 3.3	41.8 ± 2.6
Cluster 1	7.6 ± 3.3	66.2 ± 3.1	134 ± 3.2	91.1 ± 3.3	35.1 ± 2.3
Cluster 2	9.8 ± 3.1	58.5 ± 3.1	142.1 ± 4.2	85 ± 3.9	46.6 ± 3.1
Cluster 3	6.1 ± 2.5	68.7 ± 2.7	132.8 ± 3.8	96.5 ± 3.1	30 ± 3.7
Cluster 4	8.6 ± 3.5	68.5 ± 4.3	125.8 ± 2.5	91.2 ± 2.4	32.9 ± 2.5
Cluster 5	5.8 ± 2.7	64.8 ± 2.1	142.6 ± 3.1	91.7 ± 3.4	38.1 ± 2
Cluster 6	8.7 ± 2.8	64.2 ± 2.6	125.3 ± 4	86.4 ± 2.1	37.6 ± 2.3
Cluster 7	10.3 ± 2.5	54.1 ± 2.7	142.5 ± 8	74.7 ± 3	58.2 ± 7.9
Cluster 8	7.2 ± 2.6	71.9 ± 5.1	115.5 ± 3	88.9 ± 3.4	29 ± 3.5
Cluster 9	4.2 ± 2.5	73.8 ± 2.1	124 ± 4.4	94.7 ± 3	25.6 ± 2

.

Supplementary Methods S2. Data Preprocessing and Outlier Detection

Prior to analysis, data preprocessing was performed to ensure robustness of the machine learning and clustering procedures. Outlier detection was conducted using a density-based spatial clustering of applications with noise (DBSCAN) algorithm. Based on the selected parameter configuration, 100 observations were identified as outliers and excluded from subsequent analyses. This approach allowed for the removal of isolated samples that did not conform to the overall data density structure, thereby reducing potential noise effects in downstream analyses.
